# Anterior/Posterior Competitive Deactivation/Activation Dichotomy in the Human Hippocampus as Revealed by a 3D Navigation Task

**DOI:** 10.1371/journal.pone.0086213

**Published:** 2014-01-27

**Authors:** Isabel Catarina Duarte, Carlos Ferreira, João Marques, Miguel Castelo-Branco

**Affiliations:** 1 Brain Imaging Network Grid, ICNAS, Portugal; 2 Institute for Biomedical Imaging and Life Sciences, University of Coimbra, Coimbra, Portugal; University of New England, Australia

## Abstract

Anterior/posterior long axis specialization is thought to underlie the organization of the hippocampus. However it remains unclear whether antagonistic mechanisms differentially modulate processing of spatial information within the hippocampus. We used fMRI and a virtual reality 3D paradigm to study encoding and retrieval of spatial memory during active visuospatial navigation, requiring positional encoding and retrieval of object landmarks during the path. Both encoding and retrieval elicited BOLD activation of the posterior most portion of hippocampus, while concurrent deactivations (recently shown to reflect decreases in neural responses) were found in the most anterior regions. Encoding elicited stronger activity in the posterior right than the left hippocampus. The former structure also showed significantly stronger activity for allocentric vs. egocentric processing during retrieval. The anterior vs. posterior pattern mimics, from a functional point, although at much distinct temporal scales, the previous anatomical findings in London taxi drivers, whereby posterior enlargement was found at the cost of an anterior decrease, and the mirror symmetric findings observed in blind people, in whom the right anterior hippocampus was found to be larger, at the cost of a smaller posterior hippocampus, as compared with sighted people. In sum, we found a functional dichotomy whereby the anterior/posterior hippocampus shows antagonistic processing patterns for spatial encoding and retrieval of 3D spatial information. To our knowledge, this is the first study reporting such a dynamical pattern in a functional study, which suggests that differential modulation of neural responses within the human hippocampus reflects distinct roles in spatial memory processing.

## Introduction

Previous studies across several species have investigated the neuronal correlates of encoding and retrieval of spatial information, with an emphasis on hippocampal circuits and in particular their functional parcellation in rodents [1]. It is also widely accepted that the rodent hippocampus works as a cognitive map [2], thus underlying spatial memory and navigation.

In humans, the hippocampus plays a key role in different aspects of memory formation [3]–[6], and is well known to show hemispheric specialization in terms of visual [7], [8] and verbal material [9]. Less is known about the topic of hippocampal long-axis specialization. Studies of memory processing using face/name associations found functional differences within anterior and posterior regions in the hippocampus. Accordingly, the anterior hippocampus was suggested to be associated with encoding processes, while its posterior portion was linked to the retrieval of associative memories [10], [11]. There is also converging evidence for anterior hippocampal involvement in emotion processing and novelty detection [12], [13]. Direct connectivity patterns with the amygdala are also consistent with this evidence [14].

It is therefore well established that the anterior and posterior hippocampus do differ in which concerns their functional properties [12] and that the posterior hippocampal is involved in spatial memory. However, it remains unknown whether such differences in the hippocampal long-axis are associated with differential and even antagonistic neural processing patterns. A few structural studies on spatial memory and navigation suggest that this might indeed be the case. Accordingly, in the notable structural study of the hippocampus of London taxi drivers [15], the posterior volume of this structure was significantly larger relative to those of control subjects who were not taxi drivers. On the contrary, the anterior hippocampal volume was decreased in taxi drivers as compared with non taxi drivers [15]. Another remarkable example is a study of blind subjects' hippocampi [16] showing that anterior regions in the right hippocampus were significantly larger, at the cost of a smaller posterior hippocampus. To our knowledge, no functional study had so far reported that these asymmetries also hold true at a functional level and smaller temporal scale.

When studying human spatial memory it is advantageous to use realistic 3D navigational displays, allowing research on dynamic navigation with a realistic sense of size, depth and distance between objects. This is also important when studying the neural correlates of cognitive maps in terms of allocentric vs. egocentric processing, which allows to also investigate the proposed bias for allocentric spatial memory processing in the hippocampus [17].

Based on the evidence of the above mentioned studies, we hypothesize that an antagonistic functional dissociation is present between the anterior and posterior hippocampus. To test it, we studied the recruitment of hippocampus in the processes of navigational/spatial memory in normal subjects using either egocentric or allocentric strategies in the retrieval of spatial representations. The study of both encoding and retrieval within the spatial memory domain was done during active navigation and using stereoscopic vision. This 3D navigational paradigm used was kept simple to reduce the novelty factor in each trial, since novel items could potentially activate the anterior hippocampus [13]. The subject actively navigated through the scene and got a naturalistic sense of depth and object location.

Our study suggests the existence of antagonistic coupling between negative (known to be associated with decreased neural activity) and positive BOLD responses in the anterior and posterior hippocampus, suggesting that there are differential and opposed mechanisms underlying spatial memory processing in the hippocampus.

## Methods

### 2.1 Subjects

Fifteen subjects completed voluntarily the study (eight females and seven males, aged from 20 to 31 years, mean age 25.2±3.1 years). All participants are right-handed and had normal or corrected to normal vision. All subjects signed the informed consent and the study was approved by the Ethics Committee of the Faculty of Medicine of the University of Coimbra.

### 2.2 MRI experiment

Structural MRI scans were acquired in a 3T MRI scanner (Siemens Magnetom Trio Tim, Erlangen, Germany), using a 12-channel head coil. A T1-weighted MPRAGE anatomical volume was measured with repetition time (TR) of 2530 ms, time (TE) of 3.42 ms, resolution 1 mm^3^, flip angle of 7°, matrix size 256×256, field of view of 256×256 and a slice thickness of 1 mm.

### 2.3 fMRI experiments

Considering the hippocampus as one of the main structures of interest in the present study, we were aware of the susceptibility artifact. To reduce it close to hippocampus, we used an EPI sequence with reduced voxel size (2 mm^3^) and 33 slices acquired parallel to the hippocampal axis. Two runs were acquired during 13.5 minutes each, with TR 3000 ms, TE 30 ms, flip angle of 90°, matrix size 128×128 and FOV of 1536×1536.

The virtual environment was rendered through stereoscopic glasses generating 3D virtual-reality presentations (Avotec Inc., Stuart, USA) with FOV of 30 degrees horizontal and 23 degrees vertical, and a frequency of 60 Hz. The two displays present slightly different images creating binocular disparity, which creates a sense of depth, with vivid distance and size perception of objects. The subject could actively navigate through the virtual space using an MR-compatible joystick (Mag Design and Engineering, Redwood City, USA).

### 2.4 Navigational memory task

**Figure 1:**
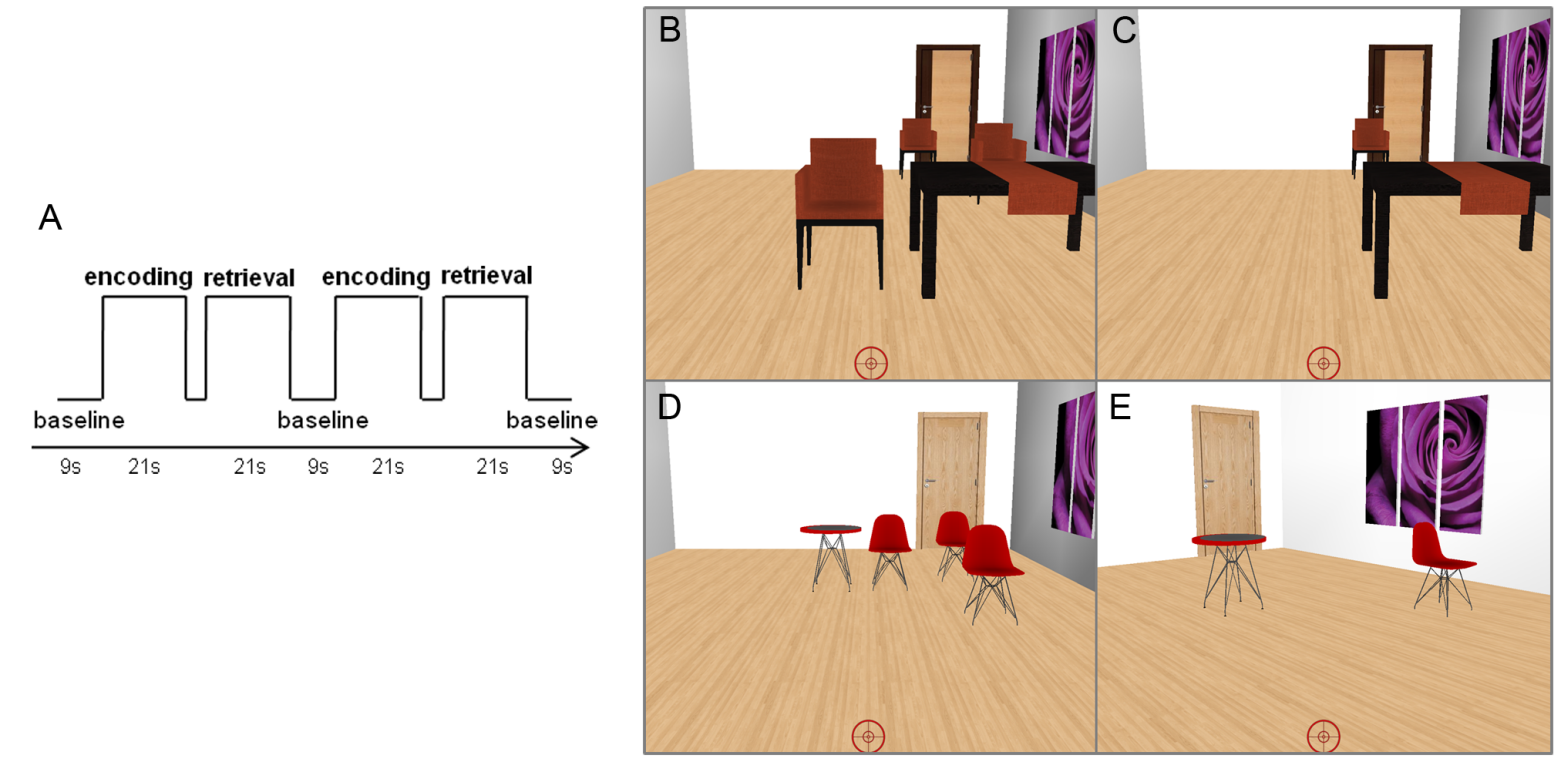
Experimental design and two scene examples. The paradigm consisted of pairs of encoding/retrieval blocks separated by a dark image during 9 seconds (A). jit – period of variable durations (3, 6 or 9 seconds). The task scene comprised a room with targets (chairs) and landmarks (door, table, picture) (B–E). During the encoding phase (B and D) the subject had to virtually move using a joystick, and click all target object locations. During the retrieval periods (C and E) the subject had to virtually move and click at the locations corresponding to the missing targets. The subject started from the same position and with the same angle of vision as in encoding when the retrieval is done in the egocentric mode (C). During the allocentric retrieval (E), the starting position and the angle of vision is changed in relation to the previous egocentric block.

The subjects complete two functional scans during the task. The navigational memory task comprised a boxcar-based design, consisting in 28 blocks of 21 seconds per functional scan. These 28 blocks of task in each run are divided in 14 blocks for memory encoding and 14 blocks for memory retrieval. After the experiment (two runs) the subject completes 28 pairs of encoding/retrieval blocks. Each encoding block is followed by the respective retrieval block, and the interval between them is jittered and can last 3, 6 or 9 seconds (Figure 1A). The baseline condition occurs between each pair of encoding/retrieval and it lasts nine seconds. Each scene of an encoding/retrieval pair has a specific set of chairs and tables, and respective positions, different of any other to minimize confusion with anterior scenes. Figure 1 (B to E) shows the encoding and the retrieval of two scene examples: a room with tables, picture, door (the landmarks) and chairs (the targets). The target chairs, which position the subject has to memorize, are presented only in the encoding phase. The door and the picture in the wall are equal in all scenes and therefore their positions remain the same along the task. The virtual environments were created using the virtual-reality toolkit Vizard (WorldViz, Santa Barbara, USA).

During encoding, the subject was instructed to navigate towards the door and along the path to click (with the joystick button) in the chairs (and therefore in their respective position) to “remove” these objects as the participant memorizes their position. The number of targets to memorize varied and it could be 3, 4 or 5 chairs. In the retrieval phase, the scene reappeared and only the landmarks could be seen. The subject was instructed to navigate towards the door again and click in the position of the missing chairs. In half of the scenes, the starting position and the angle of vision in the retrieval phase was different from the ones in the encoding phase to preclude the subject to restore the same frame of reference, as we did in a previous study [18]. The differences in the starting positions ranged between 1.45 and 2.75 meters to the left or right side (the virtual room mimics a 5×8.5 meters room) and the angle of vision ranged between 15 and 40 degrees (it stayed fixed during the whole block) simulating a lateral movement of head. No changes were implemented, as required, in the egocentric retrieval (Figure 1 B and C), while both types of alterations (starting position and angle of vision), were implemented during allocentric retrieval (Figure 1 D and E). In sum, half of the retrieval blocks were performed requiring egocentric representations and the other half required the use of allocentric representations.

### 2.5 Behavioral data

Measures of spatial memory performance, as indexed by the ability to respond correctly to the position of missing targets were obtained in every subject. The response (the click in the position of missing targets) was considered a hit if it occurred inside a square area centered in the chair center and the admissible margin was half of the chair length around the chair. This task was first tested in a prior study in ten other participants to adjust the difficulty level.

### 2.6 fMRI data analysis

Functional data were pre-processed and analyzed using BrainVoyager QX 2.3 (Brain Innovation, Maastricht, The Netherlands). Pre-processing included scan time correction (cubic spline interpolation), 3D motion correction (interpolation done combining trilinear and sinc methods), and filtering in the time domain (using a GLM approach with Fourier basis set, 2 cycles per time course). The anatomical and functional data were co-registered (and manually verified) and then normalized to the Talairach space [19]. A random effects (RFX) analysis was done at group level using a General Linear Model (GLM) approach and the predictors model was obtained by convolution of the time course belonging to each condition with a two-gamma hemodynamic response function. Statistical maps were corrected for multiple comparisons using cluster threshold levels with a fixed P value of 0.05 and voxel extent, which estimation was based on Monte Carlo simulations (1000 iterations). Significant clusters include at least 30 contiguous voxels.

### 2.7 ROI based Random Effects Analysis

The regions-of-interest (ROIs), left and right hippocampus, were automatically segmented using FreeSurfer 5.1 (http://surfer.nmr.mgh.harvard.edu) which procedure is based on an atlas of probabilistic information computed from a manually labelled dataset [20], [21] based on the Duvernoy atlas [22]. The hippocampi of all subjects were transformed into the Talairach space and were combined to perform the RFX analysis in BrainVoyager QX 2.3 (Brain Innovation, Maastricht, The Netherlands).

## Results

### 3.1 Behavioral data

All the participants performed the task at an acceptable level: the percentage of correct responses ranged from 61.8 to 86.8%; and average percentage of participants' hits was 78.1% (SD = 7.3). Considering the number of responses per target, we obtain a percentage of hits ranging between 60.1 and 86.5% (average 73.9%, SD = 7.4). Considering the two types of retrieval, the percentage of hits was 77.4% and 78.8% for egocentric and allocentric retrieval tasks, respectively.

### 3.2 General Random Effects Analysis

Although the focus of this study was the hippocampus we also performed whole-brain group level RFX-GLM analysis (RFX, −2.14<t(14)<2.14, P<0.05, corrected) related to encoding and retrieval of spatial information during active navigation. The contrast between encoding and retrieval vs. baseline evidenced positive clusters comprising bilaterally the superior parietal lobe, occipital cortex, cuneus, as well as the right retrosplenium. Positive BOLD changes were also found in the lingual gyrus, fusiform gyrus and the posterior portion of the parahippocampal cortex. Importantly, significant increases in the BOLD signal were found involving bilaterally the posterior portion of hippocampus. Additionally, significant activity modulation was found in the thalamus, globus pallidus and putamen. Clusters of negative BOLD changes were also evident. One posterior cluster involved the posterior cingulate and the precuneus. Another one, also found bilaterally, involves the inferior and middle temporal cortex. Likewise, the medial prefrontal cortex showed significant BOLD signal decreases.

The contrast of encoding vs. retrieval (RFX, t(14) = 2.14, P<0.05, corrected) showed a cluster involving the occipital cortex and the fusiform gyrus. The enhanced BOLD signal for encoding also extends to the parahippocampal cortex. A decrease in BOLD activity was found in the posterior cingulate cortex. The right anterior hippocampus showed evidence for larger signal amplitudes for encoding than for retrieval (suggesting less deactivation in the former, see also below).

### 3.3 ROI-based Random Effects Analysis

**Figure 2:**
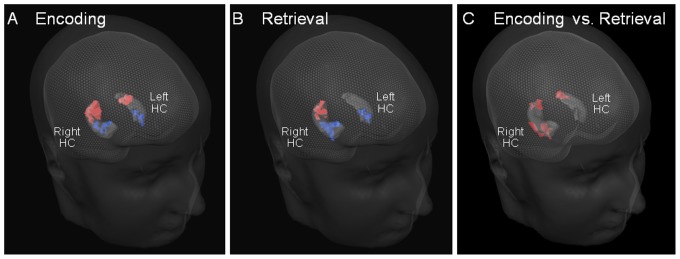
Posterior hippocampal recruitment in spatial encoding and retrieval tasks. ROI-based RFX analysis. Positive (red) and negative (blue) clusters in the hippocampus were identified in a group-level RFX analysis (P^corr^<0.05), obtained during encoding (A), retrieval (B), and contrasting encoding vs. retrieval (C).

ROI based RFX analysis provided additional evidence for a dichotomy between the anterior and the posterior portions of hippocampus in both hemispheres, during spatial navigation. Contrast analyses showed a BOLD bilateral deactivation of the anterior portion of hippocampus, while activations were found in its posterior region (P<0.05, corrected for multiple comparisons): first, this anterior/posterior deactivation/activation pattern was elicited during encoding vs. baseline (Figure 2A). Second, during retrieval vs. baseline, this pattern was found in the right hippocampus, while in the left hippocampus only the anterior negative cluster was found (Figure 2B). The left posterior positive cluster is not evident after correction for multiple comparisons in the random effects analysis. Finally, Figure 2C shows the contrast encoding vs. retrieval, which was significant and positive in the right anterior hippocampus, and bilaterally in the posterior hippocampus. Taken together these results suggest an enhanced posterior activation for encoding bilaterally, and an enhanced right anterior deactivation that was stronger for retrieval.

### 3.4 Event-related analysis of task condition

Human anterior and posterior hippocampal sub regions do not have yet a stated convention for anatomical segmentation as it exists for rodents [12]. As we have automatically segmented images in the Talairach space, we anatomically divided this structure in three subsections with an equal extent with respect to the Y axis, to further dissect the putative functional parcellation of the human hippocampus. We did then compare in detail the % BOLD signal change between the anterior most and the posterior most portions. The percentage of BOLD change was computed from the intensity fluctuations within the time window between the first and the sixth acquired volumes after the beginning of the block. The average baseline of each condition is calculated using the two previous acquired volumes, just before the beginning of the block. In this analysis we considered the two types of allocentric and egocentric retrieval separately.

**Figure 3:**
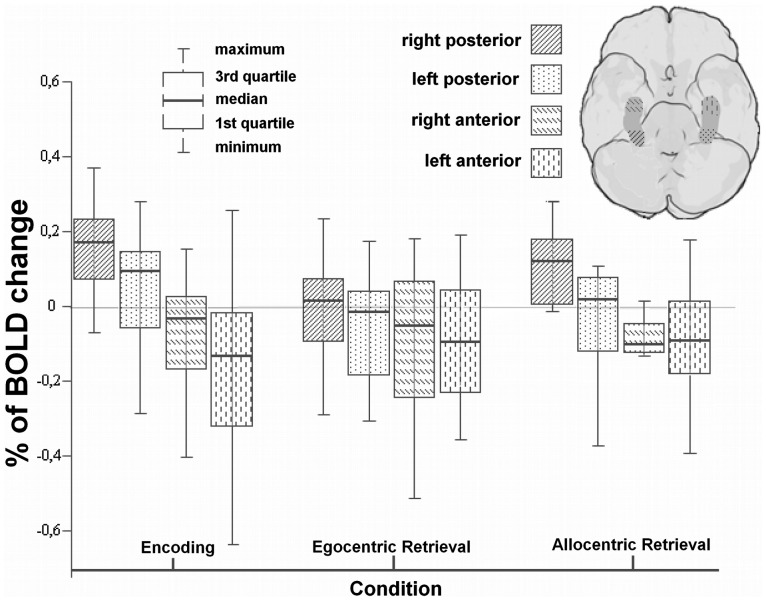
Hippocampal responses (% BOLD change) during visuospatial encoding and retrieval are polarized and dominate in the most posterior regions. Percentage of BOLD change during task performance is shown for the 4 subregions (posterior most (last third) and anterior most (first third) regions in left and right hippocampus). For statistical comparisons, see text.

Results shown in Figure 3 replicate and extend the GLM RFX findings. The anterior/posterior antagonistic pattern of BOLD activation was found to be statistically significant for encoding (Wilcoxon signed-rank test, P = 0.001 for right and P = 0.002 for left hippocampus) and for allocentric retrieval (WSR test, P = 0.001 for right and P = 0.027 for left hippocampus). No significant differences between anterior and posterior portions of the hippocampus were found during the egocentric retrieval.

Furthermore we also found that the right posterior hippocampus activates more for encoding of spatial information than for its allocentric (WSR test, P = 0.013) or egocentric retrieval (WSR test, P = 0.003). In the left side, posterior hippocampus also showed enhanced activation for encoding comparing with retrieval (WSR test, P = 0.036) or egocentric retrieval (WSR test, P = 0.005). During encoding, the percentage of BOLD change is larger in the right than in the left posterior hippocampus (WSR test, P = 0.041). Finally, we found that during 3D navigation the right posterior hippocampus activates more for allocentric than for egocentric retrieval (WSR test, P = 0.023).

## Discussion

In the present study, we tested the hypothesis of existence of antagonistic activity coupling in the anterior and posterior hippocampus, by using random effects analysis of brain activity while subjects engaged in short blocks of navigation through virtual reality rooms. We set out to compare encoding and retrieval phases of egocentric and allocentric navigation. We did indeed find an antagonistic activity pattern concerning the engagement of anterior and posterior portions of the hippocampus, providing evidence for tight antagonistic coupling between negative (known to reflect decreased neural responses [23], [24], see below) and the positive BOLD responses. These opposite patterns of neuronal activity in healthy humans lead to the interesting speculation that if sustained they might lead to long term plasticity. The posterior hippocampus was more engaged during encoding and during allocentric retrieval.

### 4.1 Asymmetry patterns concerning visual spatial encoding and retrieval

In this study we found that the posterior third of the hippocampus is involved in 3D visual spatial encoding and retrieval of object position in a path, while the anterior hippocampus concomitantly deactivates. Activation during encoding was significantly larger than retrieval, but the spatial pattern was similar. These findings were observed both for whole brain and ROI-based analysis. Moreover, the former showed a network related to 3D spatial navigation and comprising the superior parietal lobe, occipital cortex, cuneus, and right retrosplenium, that is been suggested to play a role in spatial memory [25], [26]. Positive BOLD changes were also found in the lingual gyrus, fusiform gyrus and the posterior portion of the parahippocampal cortex.

### 4.2 Negative BOLD as a stimulus related response: implication in the neuronal mechanisms underlying spatial memory

In the present study, we found a bilateral negative cluster in the anterior hippocampus that was antagonically coupled to the positive findings in its posterior most region.

The mechanisms underlying BOLD signal changes are complex and reflect a link between cerebral blood flow, energy demands and neural activity. In general one would expect that an experimental condition, e.g. motor activity or visual stimulation, would result in an increase in the BOLD signal when compared with a control condition. However, some cognitive tasks may result in a decrease in the BOLD signal [23], [27]–[30]. While the most of current fMRI research focus on the positive BOLD response, since the relation between positive hemodynamic response and the increases in neuronal activity is better characterized, little attention has so far been paid to negative BOLD response. The origins of this negative effect have been debated in terms of whether being caused by reduced neuronal input, vascular blood steal or by functional deafferentation [23], [31], [32]. However, recently, two landmark studies [23], [24] suggest a clear functional interpretation of negative BOLD, where the responses reflect suppression of neuronal activity. Shmuel et al. showed indeed that the negative BOLD response found beyond the stimulated regions in monkey visual cortex was coupled to decreases in neuronal activity below spontaneous baseline activity, rather than a purely vascular origin [23], [28]. Liu et al. also found the suppression of neuronal activity as the origin of negative responses in frontal, somatosensory and occipital regions during a finger tapping task [24]. These results show that is important to consider the functional meaning of negative responses as well the antagonistic coupling between negative and positive responses. These notions provide a more comprehensive understanding of neuronal mechanisms underlying information processing. The clusters we found in hippocampus provide evidence for antagonistic coupling between the negative and the positive BOLD responses and shed light on the neuronal mechanisms underlying spatial memory processing within hippocampus. We do believe that this antagonistic pattern has either not been noticed or gone unreported in previous spatial memory studies. We argue that other functions related to associative and episodic memory recruit the anterior hippocampus, in contrast with spatial memory. Iaria et al. focused their study on the complementary functional contributions of retrosplenium and hippocampus during spatial memory. Interestingly, during the use of spatial information, a right anterior negative cluster was present in their data. Unfortunately this finding was not discussed given that this study did focus on the anterior-posterior functional differences [33]. The complex design of the environments and the amount of novel spatial information may lead to increases of BOLD signal in the anterior regions of hippocampus as well as its associative memory processing demands [10]–[13]. Our simple paradigm and the use the same virtual room in all blocks (only changing the objects which positions subjects have to memorize) may have led to a more spatial memory isolating paradigm. This design allowed us to identify a distinct neural pattern in the anterior hippocampus during spatial memory processing, as revealed by the negative cluster signifying reduced neural activity [23], [24].

Given the scenario where this negative BOLD is considered as a correlate of neuronal activity suppression effect, it suggests that there are neuronal mechanisms common to every healthy human, showing that the possibility of global reorganization within hippocampus may vary as function of experience and therefore of neural activity, as we discuss bellow.

### 4.3 An Anterior/Posterior dichotomy in the hippocampus

The observed effects found could be generalized for the population, as demonstrated by the random effects analysis which highlighted an intriguing dichotomy between the anterior and the posterior portion of the hippocampus, bilaterally. While the posterior part of this structure increases the BOLD signal during spatial encoding and retrieval, its anterior part decreased activity for both conditions. This suggests that the anterior hippocampus is not strictly involved in spatial tasks, or at least there may be a mechanism of suppression of neuronal activity during such type of processing. Interestingly, the right anterior hippocampus yielded a positive contrast when encoding was directly compared with retrieval. This finding may seem puzzling on at first sight, because it is not due to anterior activation during encoding. In fact, anterior deactivation is smaller for encoding than retrieval, which led to the identification of such a positive contrast. In any case, an anterior vs. posterior functional dichotomy is present for both conditions.

To our knowledge this is the first functional study suggesting the existence of antagonistic coupling between the negative and the positive BOLD responses in the anterior and posterior hippocampus during spatial memory processing. Our study is consistent with a previous well-known anatomical study performed in London taxi drivers [15], though our functional paradigm tests spatial memory at a much shorter memory time scale and therefore is not a direct correlate of that study. Maguire et al. showed structural differences in the hippocampus associated with navigation experience. A pixel-counting technique was used to detect morphological changes between taxi drivers and non taxi drivers. Significant increases in gray matter volume were found in the posterior hippocampi of taxi drivers compared with those of controls, at the cost of anterior decreases. The identified posterior increases and anterior decreases in the hippocampal volume of taxi drivers as compared with non taxi drivers [15] are consistent with our functional findings, even when the time scale differences in terms of processing are taken into account. This pattern was found bilaterally and supports the idea that the posterior hippocampus is specifically involved in storage and access to 3D spatial representations of object location required for navigation. Our data put in context the recent suggestion that the anterior hippocampus is not required for precise spatial behavior, but more for context retrieving [34]. The anterior negative deactivation suggest a decrease in the neuronal activity [23], [24] that is coupled to the increases in the posterior hippocampus for strictly spatial tasks.

Another study that is consistent with our view on antagonistic patterns within the hippocampus is a study of blind subjects [16]. Major differences were found in the right hippocampus, where anterior regions showed to be significantly larger, at the cost of a smaller posterior hippocampus [16]. These findings, can be at least indirectly related to underuse of visual spatial memory, i.e. we assume that the decreased volume happens because visuospatial skills are not required, and neural activation might be chronically reduced in the posterior hippocampus in this case. These results are mirror symmetric to Maguire et al. findings which are consistent with the same interpretation. Leporé and colleagues suggest that the changes in hippocampal volumes of the presence or absence of a cognitive map and it relation to visual memories, and the possible implications for long term changes in hippocampal volume. We did find this a dynamical pattern during a 3D spatial navigation task, which can be conceptually related to the findings of Leporé in spite of the distinct temporal scale of neural changes. Moreover, the anterior/posterior amplitude difference was stronger for allocentric (and in particular for encoding) than for egocentric representations in the right hippocampus, meeting the suggestion from Leporé et al. that blind subjects do not store allocentric spatial representations, given the posterior atrophy.

It has also been assumed that spatial memory processing is lateralized to the right hemisphere [7], [8]. This is consistent with our observation of right posterior dominance in our task containing highly specific visual content and 3D navigational requirements. Nevertheless, the greatest difference was found between the anterior and posterior hippocampus, rather than between right and left.

### 4.4 Encoding vs. Retrieval: event-related analysis

The present study clearly points to a posterior hippocampal engagement in the encoding and retrieval of spatial memory during realistic 3D navigation (at the cost of anterior deactivation). Nevertheless, the results of the event-related analysis showed that despite the dual posterior hippocampal engagement during encoding and during retrieval, differences in BOLD activation were larger for encoding than for retrieval, for both hemispheres. Moreover we also confirmed a right hemispheric dominance of the posterior hippocampus in visuospatial encoding of 3D information.

### 4.5 Egocentric vs. Allocentric frames of reference

Our task design also enabled the study of the role of allo vs. egocentric frames of reference in hippocampal processing during retrieval. We did find higher recruitment of the right posterior hippocampus during allocentric vs. egocentric processing during retrieval. Allocentric and egocentric representations were previously thought to be distinct and dissociated processes [17], [35]. Our data is partially consistent with this view but does not exclude a scenario where they can be combined [36], [37]. The present study does nevertheless support the notion that allocentric retrieval is a process that dominates in the right posterior hippocampus [38].

This bias for allocentric processing in the posterior hippocampus during retrieval is interestingly consistent with the hypothesis that blind individuals, when compared with sighted subjects, have better performance in navigational tests when using egocentric frames of reference [39]–[41] and the above mentioned structural study. Our results do not show an anterior/posterior antagonistic pattern during egocentric retrieval, but just during allocentric retrieval. This is a skill not required by blind people, who show a symmetric structural antagonistic pattern [16].

### Conclusion

In sum, we have found a particular form of functional dichotomy between the anterior and posterior hippocampus, which is suggestive of existence of antagonistic coupling between the negative (reflecting decreased neural activity [23], [24]) and the positive BOLD responses. The former deactivates while latter is recruited during storage and retrieval of 3D spatial navigation information. This antagonistic pattern suggests a dynamical modulation of activity that sheds light on the neuronal mechanisms underlying the spatial memory processing within hippocampus. While the posterior positive activations corroborate the idea that the posterior hippocampus is specifically involved in storage and access of spatial memory, the anterior deactivations suggest dynamical coupling given the high processing requirements of the posterior hippocampus during the spatial memory task.

These findings extend, albeit at a shorter time scale, previous anatomical work in taxi drivers and blind subjects and suggest a dominant role of the right posterior hippocampus in 3D spatial navigation, in particular during encoding and allocentric retrieval. The evidence for activity suppression found in this study suggests cross regional inhibition, an issue that should be explored in future studies.
